# Fibroscan for assessing liver fibrosis: An acceptable alternative for liver biopsy: Fibroscan: an acceptable alternative for liver biopsy

**Published:** 2011-03-01

**Authors:** Reza Malekzadeh, Hossein Poustchi

**Affiliations:** 1 Digestive Disease Research Center, Shariati Hospital, Tehran University of Medical Sciences, Tehran, IR.Iran

**Keywords:** Fibroscan, Liver fibrosis, Diagnosis, Liver biopsy

There is now adequate evidence to indicate that liver fibrosis is a dynamic rather than a static process and as effective antiviral and other specific therapies became available, fibrosis and even cirrhosis could become reversible [1][2]. Effective and early therapy of viral and autoimmune hepatitis could result in reversibility of cirrhosis in addition to clinical cure [1][2][3]. Therefore, we urgently need to be able to follow the progression or regression of fibrosis in response to therapy in addition to initial assessment of fibrosis by liver biopsy. Liver biopsy which was first introduced in 1923 has been used widely in the diagnosis of liver diseases and is still the gold standard reference for the assessment of liver fibrosis during the course of chronic liver diseases [4][5]. But this procedure is costly, painful and runs a small risk of sever complications like hemorrhage and even death; therefore, it is unacceptable to patients and doctors alike [5]. It is also time-consuming and labor-intensive; the assessment is subjective and due to small size of the specimen in setting of heterogeneity of liver fibrosis is prone to sampling error. There is now increasing evidence that sampling error (up to 35%), inter- and intra-observer variability (up to 20%) for a particular stage between pathologist are major problems which may preclude accurate fibrosis staging for individual patients with liver biopsy [4][5][6]. For the said reasons and especially in chronic liver disease where monitoring the evolution of disease or response to treatment may require repeated assessments, the discipline of hepatology need to find a reliable and noninvasive alternative method to replace this old procedure. Finding a noninvasive method for assessment of liver fibrosis has become a real challenge for hepatologists during the last two decades. This is especially true given that chronic liver diseases affect hundreds of millions of people worldwide, the majority of which living in Asian countries [7][8][9].

Several methods have been proposed to noninvasively stage liver fibrosis including a variety of imaging modalities and a range of biochemical tests [10][11][12]. The blood tests or their composite scores like Fibro Test or aspartate aminotransferase to platelets ratio index (APRI) [11] or serum fibrosis marker such as hyaluronic acid [12] have been shown to be of limited diagnostic value especially in HBV-related chronic liver disease. The imaging modalities include ultrasound-based transient elastography (TE) [10], magnetic resonance elastography [13] and fibrocomputed tomography [14]. According to available evidence fibrocomputed tomography and magnetic resonance elastography which require the involvement of a radiologist with extensive training and a lot of financial investment, do not seem to have clear advantages in staging fibrosis when compared to TE.

In this issue of Hepatitis Monthly, Sporea and colleagues [15] used fibroscan to measure liver stiffness and estimate the extent of fibrosis in subjects with active and inactive chronic HBV infection. They have shown that patients with active HBV infection and those with an elevated HBV-DNA load have significantly higher fibroscan score compared to those with inactive HBV. Although their study have several shortcomings including significantly older age of the control group patients, absence of viral markers and liver function test measurements and sonography in the control group, absence of liver biopsy information especially on those with a higher fibroscan score and absence of estimation for visceral adiposity by waist-to-hip ratio or waist circumference measurement, their finding was similar to a previous study particularly in Asian population which indicates that TE could be used at least as a screening test to select the chronic HBV-infected individual with normal or minimally elevated transaminase for liver biopsy [10][16]. Using liver biopsy in 132 chronic HBV-infected subjects in Tehran [17] with persistently normal ALT, we have shown that up to 30% of them (mean age of 32 years) had a Knodell histologic stage of ≥ 2. Further studies comparing the actual histology stage with fibroscan measurment is necessary to find out the cutoff value in different pouplations. All evidece up to now is supporting that TE using fibroscan is a reliable, noninvasive method for identification of patients with significant hepatic fibrosis. TE is readily reproducible and its score has low inter- and intra-observer variability [10][16][18]. Several factors were found to affect the accuracy of TE for the diagnosis of significant fibrosis. These included sever liver congestion, hyperbilirubinemia, transaminitis, and prolonged prothrombin time. These factors were found to cause a significant overestimation of TE values, leading to false-positive identification of fibrosis [10][18]. In addition, TE is impossible in patients with ascites and is difficult in obese subjects and those with very narrow intercostal spaces. But, its use especially in chronic HBV-infected subjects with normal or minimally elevated ALT-which constitutes the majority of chronic liver disease in Asian population-to select the subject who may benefit from oral nucleoside analogue therapy is very attractive.

The accuracy of fibroscan score is excellent for the diagnosis of cirrhosis; it is probably the most accurate noninvasive method for the early detection of cirrhosis. It is a user-friendly technique that can be performed without any preparation in the less than five minutes in clinic or at the bedside, with immediate results and high patient acceptance, it is very likely that in future it will become the most widely used technique for assessment of liver fibrosis [10][18]. Further technological improvements are necessary for better application of this technique in obese patients and other specific populations along with efforts to improve and standardize the procedure and adequate operator training [19].

## References

[1] Malekzadeh R, Mohamadnejad M, Rakhshani N, Nasseri-Moghaddam S, Merat S, Tavangar SM, Sohrabpour AA (2004). Reversibility of cirrhosis in chronic hepatitis B. Clin Gastroenterol Hepatol.

[2] Malekzadeh R, Mohamadnejad M, Nasseri-Moghaddam S, Rakhshani N, Tavangar SM, Sohrabpour AA, Tahaghoghi S (2004). Reversibility of cirrhosis in autoimmune hepatitis. Am J Med.

[3] Mohamadnejad M, Malekzadeh R, Nasseri-Moghaddam S, Hagh-Azali S, Rakhshani N, Tavangar SM, Sedaghat M, Alimohamadi SM (2005). Impact of immunosuppressive treatment on liver fibrosis in autoimmune hepatitis. Dig Dis Sci.

[4] Menghini G (1958). One-second needle biopsy of the liver. Gastroenterology.

[5] Standish RA, Cholongitas E, Dhillon A, Burroughs AK, Dhillon AP (2006). An appraisal of the histopathological assessment of liver fibrosis. Gut.

[6] Mohamadnejad M, Tavangar S, Sotoudeh M, Kosari F, Khosravi M, Geramizadeh B, Montazeri G, Estakhri A, Mirnasseri MM, Fazlollahi A, Zamani F, Malekzadeh R (2010). Histopathological Study of Chronic Hepatitis B: A Comparative Study of Ishak and Metavir Scoring Systems. IJOTM.

[7] Ganji A, Malekzadeh F, Safavi M, Moghaddam SN, Nouraie M, Merat S, Vahedi H, Zendehdel N, Malekzadeh R (2009). Digestive and Liver Disease Statistics in Iran. Middle East J Dig Dis.

[8] Alavian SM, Ahmadzad-Asl M, Lankarani KB, Shahbabaie MA, Bahrami Ahmadi A, Kabir A (2009). Hepatitis C Infection in the General Population of Iran: A Systematic Review. Hepat Mon.

[9] Merat S, Rezvan H, Nouraie M, Jamali A, Assari S, Abolghasemi H, Radmard AR, Zaer-Rezaii H, Zeid-Abadi-Nejhad M, Hosseini MR,, Amini-Kafiabad S, Maghsudlu M, Pourshams A, Malekzadeh R (2009). The prevalence of hepatitis B surface antigen and anti-hepatitis B core antibody in Iran: a population-based study. Arch Iran Med.

[10] Talwalkar JA, Kurtz DM, Schoenleber SJ, West CP, Montori VM (2007). Ultrasound-based transient elastography for the detection of hepatic fibrosis: systematic review and meta-analysis. Clin Gastroenterol Hepatol.

[11] Mohamadnejad M, Montazeri G, Fazlollahi A, Zamani F, Nasiri J, Nobakht H, Forouzanfar MH, Abedian S, Tavangar SM, Mohamadkhani A, Ghoujeghi F, Estakhri A, Nouri N, Farzadi Z, Najjari A, Malekzadeh R (2006). Noninvasive markers of liver fibrosis and inflammation in chronic hepatitis B-virus related liver disease. Am J Gastroenterol.

[12] Montazeri G, Estakhri A, Mohamadnejad M, Nouri N, Montazeri F, Mohammadkani A, Derakhshan MH, Zamani F, Samiee S, Malekzadeh R (2005). Serum hyaluronate as a non-invasive marker of hepatic fibrosis and inflammation in HBeAg-negative chronic hepatitis B. BMC Gastroenterol.

[13] Huwart L, Sempoux C, Vicaut E, Salameh N, Annet L, Danse E, Peeters F, ter Beek LC, Rahier J, Sinkus R, Horsmans Y, Van Beers BE (2008). Magnetic resonance elastography for the noninvasive staging of liver fibrosis. Gastroenterology.

[14] Romero-Gómez M, Gómez-González E, Madrazo A, Vera-Valencia M, Rodrigo L, Pérez-Alvarez R, Pérez-López R, Castellano-Megias VM, Nevado-Santos M, Alcón JC, Solá R, Pérez-Moreno JM, Navarro JM, Andrade RJ, Salmerón J, Fernández-López M (2008). Optical analysis of computed tomography images of the liver predicts fibrosis stage and distribution in chronic hepatitis C. Hepatology.

[15] Sporea I, Nicolitâ D, Şirli R, Deleanu A, Tudora A, Bota S (2011). Noninvasive Liver Stiffness Assessment By Means Of Transient Elastography (Fibroscan) In Inactive Hbsag Carriers. Hepat Mon.

[16] Chang PE, Lui HF, Chau YP, Lim KH, Yap WM, Tan CK, Chow WC (2008). Prospective evaluation of transient elastography for the diagnosis of hepatic fibrosis in Asians: comparison with liver biopsy and aspartate transaminase platelet ratio index. Aliment Pharmacol Ther.

[17] Montazeri G, Rahban M, Mohamadnejad M, Zamani F, Hooshyar A, Fazlolahi A, Abedian S, Ghoujeghi F, Estakhri A, Montazeri F, Razjoyan H, Mamarabadi M, Alimohamadi M, Tavangar SM, Malekzadeh R (2010). Liver histology and HBV DNA levels in chronically HBV infected patients with persistently normal alanine aminotransferase. Arch Iran Med.

[18] Vizzutti F, Arena U, Marra F, Pinzani M (2009). Elastography for the non-invasive assessment of liver disease: limitations and future developments. Gut.

[19] Talwalkar JA (2007). Methodologic issues with transfer of ultrasound-based transient elastography into clinical practice. J Hepatol.

